# Aminopeptidase M17 in bacteria: insights into structure, function, and potential as a drug target

**DOI:** 10.1128/jb.00504-25

**Published:** 2025-12-30

**Authors:** Hussam Askar, Shengli Chen, Huafang Hao, Xiangrui Jin, Ahmed Adel Baz, Shimei Lan, Zhangcheng Li, Yuefeng Chu

**Affiliations:** 1 State Key Laboratory of Animal Disease Control and Prevention, College of Veterinary Medicine, Lanzhou University, Lanzhou Veterinary Research Institute, Chinese Academy of Agricultural Sciences 111658 , Lanzhou, China; 2 Gansu Province Research Center for Basic Disciplines of Pathogen Biology, Lanzhou, China; 3 Key Laboratory of Veterinary Etiological Biology, Key Laboratory of Ruminant Disease Prevention and Control (West), Ministry of Agricultural and Rural Affairs, Lanzhou, China; 4 Zoology Department, Faculty of Science, Al-Azhar University 68820, Assiut, Egypt; 5 Botany and Microbiology Department, Faculty of Science, Al-Azhar University 68820, Assiut, Egypt; University of Notre Dame, Notre Dame, Indiana, USA

**Keywords:** leucyl-aminopeptidase, M17, biological functions, drug target, antibiotic resistance

## Abstract

Leucyl-aminopeptidase (LAP) is a type of protease that targets peptides and the nitrogen terminus of protein molecules, playing a key role in the removal of amino acids. This function is not only significant but also enlightening, as it contributes to our understanding of microbial survival and persistence. The presence of M17-LAPs enzymes across various bacterial species indicates the possibility of creating selective inhibitors, offering new avenues for antimicrobial development amidst increasing antibiotic resistance. Additionally, understanding the relationship between the structure of these enzymes and their functions can aid in the development of more effective treatment methods and enhance current therapies. In this review, we unravel the structural blueprints, functional roles, and therapeutic promise of M17-LAPs, highlighting their relevance in the era of escalating antibiotic resistance. We also highlight future research avenues, emphasizing structural biology and protein–protein interaction mapping as keys to unlocking targeted therapeutic strategies. By bridging molecular structure with translational potential, we propose a new vision: harnessing the vulnerabilities of M17-LAPs to inspire next-generation antibacterial strategies.

## INTRODUCTION

Leucyl-aminopeptidase (LAP) is a vital metallopeptidase that facilitates the breakdown of amino acid residues from the N-terminus of proteins and polypeptides, with a preference for the peptide bond involving N-terminal leucine (1). In addition to bacteria, these enzymes have also been discovered in various organisms, including humans and plants. They are located in the cytoplasm, multiple subcellular organelles, and as membrane components. Essential cellular functions are performed by numerous aminopeptidases (2). Early research on bacterial aminopeptidases began in the 1960s, spurred by fundamental and applied interests (3), after the 1930s discovery of the first microbial aminopeptidase from *Aspergillus parasiticus* (4). *Lactobacillus*, *Bacillus*, *Actinomyces*, and numerous other microbial species have been identified as producing aminopeptidases (1). Aminopeptidases are primarily classified as metalloenzymes. One of the most abundant and consistent groups of aminopeptidases is the metalloaminopeptidases. At their active sites, these enzymes typically contain two metal ions. Histidine, glutamine, aspartic acid, and lysine are the four key conserved active sites that characterize each of the 25 families of metalloaminopeptidases. Evolutionarily, these families may be further classified as MF, MG, and MH (5). Many peptidase families utilize divalent metal ions, predominantly zinc (Zn) ions, for substrate hydrolysis at the active site. Specifically, the leucyl-aminopeptidase obtained from the bovine lens is classified within the MF family of metalloaminopeptidases, which are distinguished by the presence of two zinc ions in the active site that aid in catalysis (6). Aminopeptidases are conventionally categorized into two main types: those involved in nitrogen metabolism and those involved in protein activation or breakdown (3). Aminopeptidases in several species, primarily lactic acid bacteria, hydrolyze exogenous peptides assimilated into the cell as a nitrogen source (7). Research indicates that aminopeptidases play a crucial role in the processing of proteins produced within the organism. These enzymes cleave amino acids from the nitrogen terminus of protein chains, which is essential for both activation and inactivation processes (8–12). Previous studies indicate that aminopeptidases may significantly contribute to pathogenesis. The extracellular function of virulence-associated aminopeptidases, which are often released by various bacterial and protozoan pathogens, reveals a broader spectrum of roles for these enzymes than previously recognized (13–19). The specific residues and chemical groups that play crucial roles in catalysis and substrate selection elegantly categorize aminopeptidases into distinct functional families (20). Most recently, the M17 family of metallopeptidases has been the subject of significant research and investigation. Members of the M17 family are LAPs found in various organisms, including bacteria, plants, and humans (21). LAPs are similar at the amino acid level, with monomers (typically 53–55 kDa) assembling to form a homo-hexameric enzyme. While many LAPs primarily break down leucine residues, some are capable of working with a broader range of substrates. The recent surge in research on members of the M17 family has been attributed to their potential as candidates for therapeutic intervention and vaccine development in various protozoan and parasitic disorders. Furthermore, LAPs have emerged as intriguing players in bacteria, having been investigated in a limited number of species and demonstrating their role in virulence across numerous organisms. LAPs, found in a diverse range of bacteria including *Salmonella typhimurium*, *Escherichia coli*, *Vibrio cholera*, and *Pseudomonas aeruginosa*, do not just bind to DNA proteins with aminopeptidase activity. They also serve a critical function as transcriptional regulators, controlling the expression of genes in these bacteria (22–25). Evidence from *Bacillus thuringiensis* indicates that LAP may be involved in activating the Cyt1Aa toxin (26). M17 aminopeptidases are recognized for their proteolytic roles; however, M17 family members have been assigned a variety of other functions, in addition to aminopeptidase activity (27). In M17 bacteria, aminopeptidases attach to DNA in a precise sequence to modulate transcription (23) and create hetero-oligomers (28–30), which govern site-specific DNA recombination (24, 31).

Recent studies have shown that *pepZ* encoding LAP is crucial for the pathogenicity of *Staphylococcus aureus*. Notably, disrupting the *pepZ* gene in USA300 or Newman leads to a significant reduction in pathogenicity in both systemic and localized infection scenarios. Bacterial viability within human macrophages depends on LAP, which is located in the cytosol, and *pepZ* gene expression is at the highest level in the intracellular environment (32).

Moreover, the aminopeptidase inhibitor Bestatin significantly reduced *Mycobacterium tuberculosis* (MtLAP) activity, prevented *M. tuberculosis* from growing *in vitro*, and decreased macrophage infection. This suggests that MtLAP plays a crucial role in key metabolic pathways necessary for *M. tuberculosis* survival and virulence, making it a promising target for new *M. tuberculosis* drugs (33).

M17-LAPs, especially those from pathogenic organisms, have become a promising and validated drug target for developing new anti-infective agents. Its therapeutic value lies in its vital role during the final stages of protein breakdown, releasing free leucine and other hydrophobic amino acids essential for bacterial metabolism, growth, and stress response. In the malaria-causing parasite *Plasmodium falciparum*, the M17-LAP (PfLAP) is crucial for digesting hemoglobin within the parasite’s food vacuole, inhibiting PfLAP either genetically or chemically causes a dangerous buildup of undigested peptides, amino acid deficiency, and ultimately kills the parasite, confirming its importance as a drug target (34)

The M17 aminopeptidase family is versatile, performing a range of organism-specific roles that go beyond basic peptide hydrolysis. Yet, it remains unclear how a standard enzyme structure and reaction process can enable such diverse functions. This review examines the research on aminopeptidase M17 to elucidate the mechanisms underlying its multifunctionality.

## STRUCTURE AND FUNCTION OF THE M17 AMINOPEPTIDASE

### Variation among M17-LAPs in different species

Because bacterial genetics may be easily altered and enzyme inhibitors can be administered, the M17-LAP enzymes from bacteria have been physiologically characterized in great detail. This is due to the unique susceptibility of these enzymes to alteration and the potential for studying their physiological roles through the use of inhibitors. While the majority of bacteria have only one M17-LAP, members of the gamma proteobacteria class, such as *E. coli*, frequently carry two, known as *PepA* and *PepB* (35–37). Deletion of the *pepA* and *pepB* genes in *E. coli* produces viable bacteria (38), demonstrating that cell culture survival does not depend on either gene alone. The enzymes display distinct reaction specificities, which are 26% identical (36, 39). The most extensively researched of these proteins, EcPepA (also known as XerB and CarP), mediates site-specific DNA recombination (24, 31) and regulates transcription (23). EcPepA binds directly to the carP1 promoter to regulate transcription, inhibiting gene expression in the *carAB* operon. Carbamoylphosphate synthase is an enzyme required for pyrimidine biosynthesis; this regulatory event regulates its expression and, possibly, that of the e*cpepA* gene, suggesting negative autoregulation (23, 40). EcPepA, the primary structural component of a heterocomplex, is responsible for the intricate process of multicopy plasmids. It facilitates sequence-specific recombination that has undergone multimerization. This process, crucial for the stability and longevity of plasmid genetics, is regulated by a unique and complex DNA-binding mechanism (24, 31). Although the *pepA* DNA-binding events have two different purposes, only one DNA-binding site has been identified.

The crystal structure of the hexamer revealed an extended space along its interface, where basic amino acids are exposed to the surface, providing sufficient space to accommodate a DNA helix. Specific N-terminal residues were identified as crucial for DNA binding through concurrent mutational studies, a process guided by the reliable X-ray crystal structure (29, 30, 41). A complicated DNA sequence involves two EcPepA hexamers and one ArgR hexamer, which places both cer sites close together (27).

Researchers utilized the SWISS-MODEL and PHYRE2 programs to construct three-dimensional (3D) models of *Bacillus cereus* (BcLAP). The three-dimensional conformation of a protein, which is a crucial factor in its biological activity (1), was thus revealed. Protein structures are often predicted from amino acid sequences utilizing a variety of methodologies and software packages. A prerequisite for success is the ability to identify a homologous protein with high sequence identity in the three-dimensional structure (42). In the case of *E. coli* PepA and BcLAP, which share a 37% sequence identity and 45% similarity in their C-terminal regions, the powerful tool of X-ray crystallography has been instrumental in unveiling the monomeric and hexameric structures of EcLAP.

Researchers showed that the BcLAP monomer exhibits considerable structural similarities with EcLAP and PpLAP. Two separate domains make up the structure of BcLAP: a lengthy, conserved C-terminal section (residues 178–485) and a short, highly variable N-terminal domain (residues 15–143). This conserved nature of the C-terminal region adds a sense of stability and integrity to the structure of BcLAPs. A lengthy helical linker was found to connect the two domains. Two α-helices flank the five core β-strands that make up the BcLAP N-terminal domain. Both EcLAP and *Pseudomonas putida* PpLAP have an N-terminal domain made up of six central strands with two α-helices flanking the β-strands. The complex, triple-layered structure of the C-terminal domain of BcLAP, defined by an eight-stranded β-sheet surrounded by five α-helices on each side, is a fascinating area of study. At last, the catalytic site of aminopeptidase has been identified in the C-terminal domain, a key functional region responsible for the enzyme’s catalytic activity. Characteristically comparable to EcLAP and PpLAP is the shape of the C-terminal region of BcLAP (1).

The hydrophobic pocket of M17 aminopeptidases, a key player in substrate selectivity, is remarkably conserved. In bovine LAP, the substrate’s entry into the active site is significantly influenced by a nearby hydrophobic pocket on the monomer surface, a feature conserved in BcLAP. This pocket, housing seven highly conserved residues and two variable ones, Ser431 and Ser463, is a site of significant interest. The zinc (Zn) at the Zn1 site interacts with the carboxyl group of Glu344’s side chain via its amine, hydroxyl, and carbonyl groups, while the carbonyl and carboxyl groups of Asp342’s side chain do the same. Asp265 connects to the Zn via its side chain’s hydroxyl group. At the Zn2 site, Lys260, Asp265, and Asp283 coordinate on the zinc ion via their hydroxyl and amino groups of their side chains. Studies indicate that, during bovine LAP catalysis, the hydroxyl group of a polarized water molecule acts as a nucleophile, bridging the two metal ions and attacking the substrate molecules. Lys262 plays a key role in maintaining the enzyme–substrate complex structure.

Recent research findings suggest that a hydroxyl group is present between two zinc atoms in the enzyme BcLAP. Additionally, Lys272 was found to perform a role similar to that of Lys262 in bovine LAP. This function is facilitated by Lys272 forming a hydrogen bond between the carbonyl of the β-carboxyl group and the amino acids at the terminus of its side chain. This comparison is significant as it provides insights into the evolution and diversity of enzyme functions. Furthermore, hydrogen bonds elegantly connect Asp283 to the amino group of Lys260 through its carboxyl group, whereas Asp265, Glu344, and Asp342 interact with water molecules. The interactions enhanced the precise localization of these residues near the zinc particles (1).

**Fig 1:**
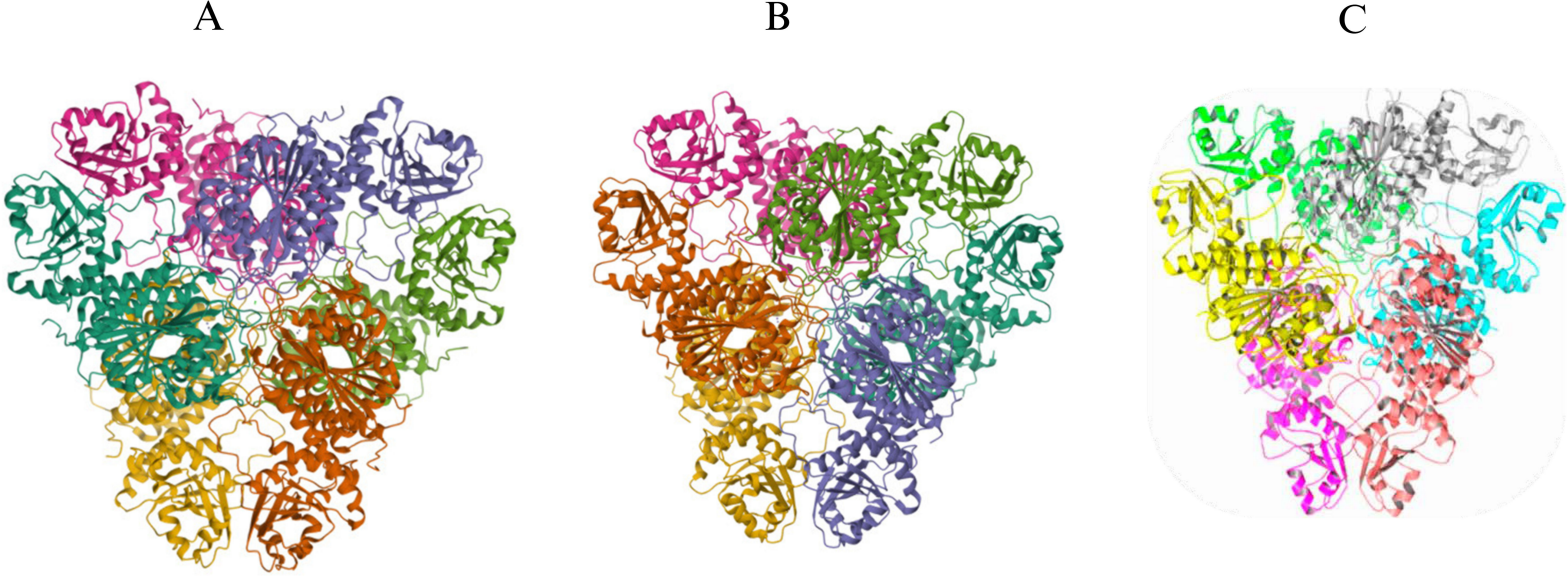
Comparison of the hexameric structures of M17-LAPs. (**A**) The EcLAP hexamer displays the canonical dimer-of-trimers organization typical of M17 enzymes. (**B**) PpLAP forms a similar hexameric arrangement with conserved catalytic pocket geometry. (**C**) BcLAP also adopts a stable hexamer, highlighting structural conservation among M17-LAPs across different bacterial species.

The study explored the intriguing role of Bestatin within the active site of BcLAP, unraveling the unique mechanisms that govern its binding and function. This was done using the hexamer of PpLAP as a reference due to the significant resemblance in the spatial arrangement of the BcLAP hexamer and PpLAP Bestatin. Recent discoveries reveal that the Bestatin-bound PpLAP and Bestatin-bound bovine lens BlLAP complexes exhibit an impressively similar binding mechanism to that of Bestatin within the BcLAP active site. This intriguing finding highlights the elegant interplay between these molecular structures (43). The findings of BcLAP and PpLAP-Bestatin reveal that in the active site of PpLAP, most residues near the two metal ions and Bestatin correspond to those in BcLAP. The comparison reveals that the key distinction between Zn1 and Zn2 is that the hydroxyl group of Bestatin replaces the cross-linking water or hydroxide ion (1). M17-LAPs display a remarkably conserved structure among different organisms, generally assembling into a hexamer with 32 symmetries. This structure is a dimer of trimers, featuring an interior cavity with six active sites and surrounding trimers (44). The BcLAP structure, carefully simulated by SWISS-MODEL, revealed traits that were strikingly similar to those of EcLAP and PpLAP (Fig. 1) and showed that the hexamer has a triangular shape when viewed along the threefold molecular axis, with a thickness of approximately 80 Å and a triangle edge length of about 135 Å. These dimensions match those of EcLAP and PpLAP, further reinforcing the familiarity of BcLAP’s structure (1). The solvent cavity at the center of the hexamer, which contains the active site of the aminopeptidase, is a significant feature. This cavity is composed of several hydrophobic amino acids, including proline, valine, leucine, serine, alanine, threonine, and glycine. The catalytic region is positioned at the threefold axis and plays a vital role in facilitating interactions among the components of each trimer unit and between the two trimers. Three passages provide access to the cavity: one is located at the double-fold molecular core, and the other pair is uniquely positioned at the connection between the nitrogen-terminal and both of the C-terminal regions. The ends of the enzyme motifs are essential for linking the two trimers adjacent to the two-dimensional axis, highlighting the enzyme’s complex and fascinating structure. The structural characteristics of BcLAP, particularly its similarity to *PepA*, provide reassurance about the enzyme’s classification (45).

A single hexamer of EcPepA, as a key player, adheres to the DNA via the nitrogen-terminal basic region, mediating the transcriptional control of the *carAB* operon, a complex process in molecular biology. By interacting with two out of the three cavities, the hexamer creates an advantageous coil structure that encircles the *carAB* promoter region around 235 base pairs (28, 39). Although its precise function is still a mystery, the heterocomplex formed by EcPepA and its auxiliary proteins plays a crucial role in building the synaptic complex between two plasmid recombination sites. This intricate collaboration highlights the elegance of molecular interactions in the realm of genetic engineering (28–30).

It is enlightening to learn that numerous bacterial species, including *V. cholerae*, have been shown to exhibit M17-LAPs transcriptional regulatory capabilities. The pH and temperature of the surrounding environment have a significant impact on the intricate circuit that controls the virulence components, including cholera toxin, in *V. cholerae*. The key factor driving the rise of cholera toxin levels under non-inducing conditions (pH 8.4 and 37°C), where these toxins are usually undetectable, is the loss of the gene responsible for *V. cholerae*, known as VcPepA. This unexpected genetic alteration highlights a fascinating aspect of cholera’s complexity and its ability to thrive under unusual circumstances (22). On the other hand, toxin levels were unaffected by the absence of VcPepA under “inducing conditions” (pH 6.5 and 30°C). Following the lead of the *E. coli* system’s proposed consensus target sequences, other investigations have located a potential target sequence inside the *V. cholerae* genome (23), suggesting that VcPepA may bind to this sequence. They propose that VcPepA regulates toxin production by modulating transcription in response to varying environmental conditions.

Additionally, *P. aeruginosa*’s PepA homolog (PhpA) regulates transcription. The *algD* gene encodes an enzyme that participates in the alginate biosynthesis pathway, while PhpA regulates transcription at this locus (46). Overproduction of alginate, a component in biofilm formation, is characteristic of the mucoid phenotype seen in cystic fibrosis lung infections (47). Research on *S. aureus* adds to the growing body of evidence that bacterial M17-LAPs have a role in pathogenicity. One cytosolic enzyme is the M17-LAP produced by *S. aureus* (SaM17-LAP) (32). While an earlier study indicated its possible secretion (48), this observation was subsequently attributed to protein A’s non-specific binding to the antibodies used in the Western blot analysis rather than SaM17-LAP (32). Thorough research, which included changing the gene (*pepZ*) and then studying the results in both lab and living models, has revealed several important discoveries. They found that SaM17-LAP is not essential for the development of *S. aureus*, suggesting that it likely does not fulfill a nutritional role. However, the enzyme’s role in bacterial virulence is of utmost importance. SaM17-LAP is primarily found in the intracellular spaces of macrophages, where it plays a crucial role in their functionality. Notably, survival tests indicate that this protein is not only important but also essential for maintaining bacterial viability in human macrophages. The *pepZ* gene is crucial to pathogenesis, as *in vivo* mouse studies have revealed that *S. aureus* lacking it exhibits significantly reduced virulence in both localized and systemic infections (32). These findings reinforce the significance of the *pepZ* gene in *S. aureus* virulence, offering valuable insights to the scientific community.

Other research has shown contradictory findings on the role of SaM17-LAP, suggesting that Bestatin therapy prevented *S. aureus* from growing and forming biofilms (48). The results are not confirmed using the *pepZ* mutant strain (49). It is fascinating to consider the possibility that these results may be caused by the off-target effects of Bestatin, a peptidase inhibitor known to block the M1 aminopeptidase, among others (50). These findings underscore the need for further research in this area.

Several studies investigating M17 enzymes in bacteria indicate that Bestatin treatment leads to growth inhibition; for instance, in *M. tuberculosis*, Bestatin suppressed *in vitro* bacterial proliferation and survival within macrophages and diminished bacterial load in murine models (33).

In periodontitis, hydrogen sulfide plays a significant role in tissue destruction, possibly generated by the breakdown of glutathione in oral bacteria. The periodontal disease-causing bacterium *Treponema denticola* is endemic to humans and is also found in the mouths of animals. By studying the bacterium’s glutathione metabolism route, scientists were able to identify an M17-LAP from *T. denticola* M17-LAP (TdM17-LAP) (51). The discovery of M17-LAP as the cysteinyl glycinase enabled the separation of cellular extracts, facilitating the second step of glutathione metabolism. The enzyme’s capacity to perform supplementary functional activities beyond glutathione catabolism remains unexplored.

*Helicobacter pylori* M17-LAP (HpM17-LAP) exhibits an enhancement in response to cellular stress induced by metronidazole and nitric oxide treatment, which also increases oxidative stress (52). Understanding this response is crucial, as these capabilities could be a result of HpM17AP’s cysteinyl-glycinase (Cys-Gly) activity (52, 53). Since Bestatin suppresses *H. pylori* growth in culture by inhibiting HpM17-LAP, one of the two aminopeptidases in the peptidase family (54).

*Mycoplasma hypopneumoniae* surface protein proteomic analysis revealed an M17-LAP (MHJ_0461, henceforth abbreviated as MhM17-LAP). MhM17-LAP, according to sequence analysis, is a cytoplasmic protein. However, it lacks any known signal sequences or membrane-binding domains; consequently, the mechanisms of export and membrane attachment are not well understood (55). The researchers demonstrated the interaction between *M. hypopneumoniae* M17-LAP and heparin, which is connected to the invasion of host cells and microbial survival within macrophages (56, 57). It also attaches to plasminogen, helping it change into plasmin, which indicates it may play a part in adjusting the body’s defense systems (55). The results of these binding assays indicate that the protein may contribute to the pathogenesis of *M. hyponeumoniae* and facilitate its continued survival within the host. The comparison to EcPepA, which shows a 26% sequence similarity, prompted an examination and subsequent confirmation of MhM17-LAP’s DNA-binding potential. This discovery could have significant and far-reaching consequences. However, MhM17-LAP’s DNA-binding technique, featuring a unique N-terminal domain DNA-binding motif, is distinct from that of EcPepA. The mechanisms underlying the DNA-binding capabilities of a surface-exposed protein remain a mystery, suggesting that this protein may have diverse functions in the cytoplasm and on the cell surface (50).

**Fig 2:**
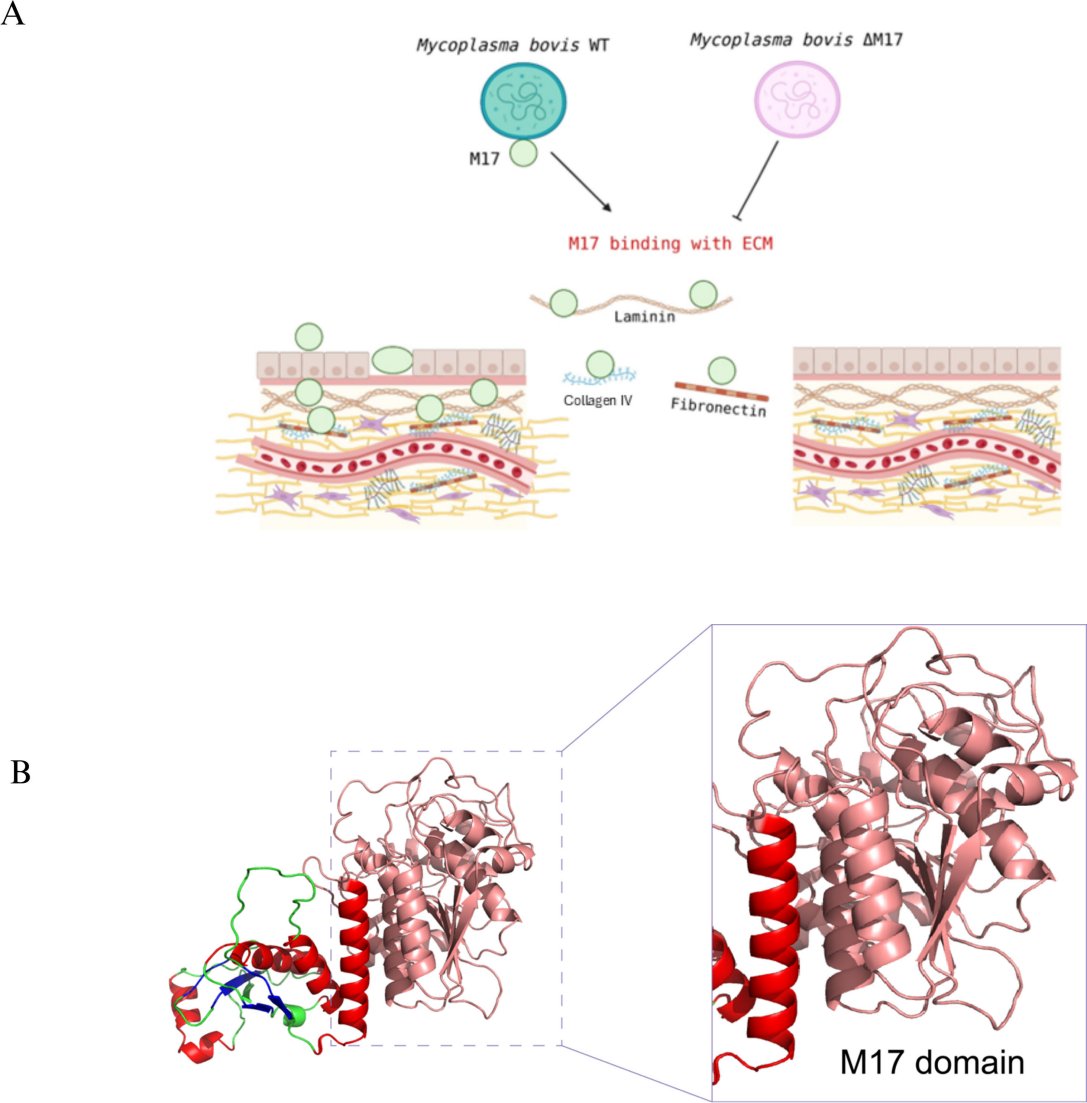
(**A**) MbM17 promotes *M. bovis* adhesion to host cells by binding to the host ECM. MbM17-LAP molecular models. I-TASSER generates a full-length protein MbM17-LAP model by extracting continuous fragments from threading alignments and then reassembling them using replica-exchange Monte Carlo simulations. (**B**) The 3D structure, including the predicted secondary structure, is shown with α-helices in red and β-strands in blue, and the M17 domain.

*Mycoplasma bovis* causes multiple inflammatory diseases in cattle. Controlling and preventing *M. bovis* infection is challenging due to the absence of effective vaccines and the emergence of multidrug-resistant strains, resulting in notable economic losses worldwide. Lipoproteins, vital components of the *Mycoplasma* cell membrane, are potent antigens that elicit immune responses during infection. However, the specific roles of lipoproteins in *M. bovis* are not well understood, as they exhibit low sequence similarity to those in other bacteria, and genetic tools for their manipulation are limited (58). Our study showed that the M17-LAP from *M. bovis* (MbM17-LAP) is present in both the bacterial cell membrane and cytosol. To further investigate M17’s ability to adhere to host cells, we tested its interaction with extracellular matrix (ECM) components. The results indicated that rMbM17 binds to ECM components in a dose-dependent manner (Fig. 2).

**Fig 3:**
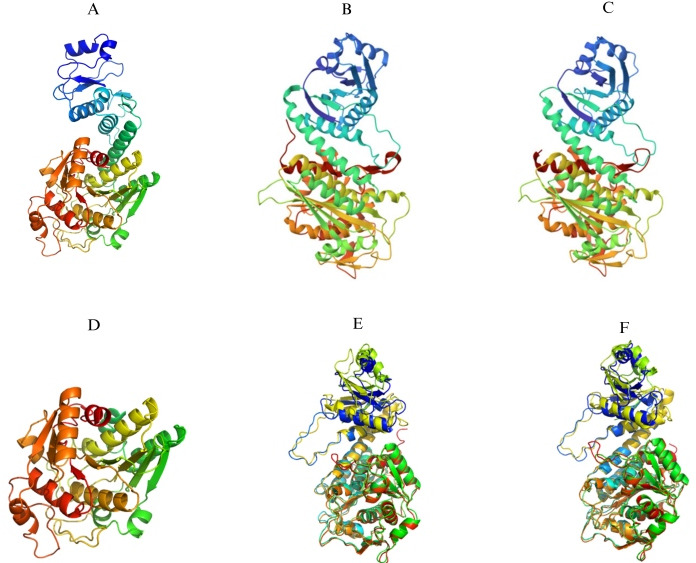
*M. bovis* MbM17-LAP molecular models generated by the I-TASSER server and PyMOL model. This comprehensive comparison includes the (**A**) MbM17-LAP monomer model, the (**B**) X-ray crystallography of EcLAP monomer N- and C-termini, and the (**C**) X-ray crystallography characterization of the N- and C-termini of the PpLAP monomer. Also included are the (**D**) PyMOL model-generated MbLAP C-terminal domain model and comparisons between (**E**) MbLAP and EcLAP, and (**F**) MbLAP and PpLAP generated by the PyMOL model.

To build 3D models of *M. bovis* MbM17-LAP, our research utilized the I-TASSER server, in conjunction with the PyMOL model application. A key component in defining the biological activity of the protein was its three-dimensional conformation, which was revealed by this procedure. Forecasting protein structures from amino acid sequences is a multifaceted endeavor that often requires the use of multiple methodologies and technologies. The success of this process hinges on accurately determining the three-dimensional structure of a homologous protein with high sequence identity. In the case of EcLAP, PpLAP, and HpLAP, which share 28%, 28%, and 25% sequence identity, respectively, and exhibit 95%, 95%, and 91% similarity in their C-terminal regions, X-ray crystallography has proven to be an invaluable tool. It has played a crucial role in uncovering the monomeric and hexameric structures of EcLAP, PpLAP, and HpLAP (Fig. 3), and its contribution to the field is significant and cannot be overstated.

**Fig 4:**
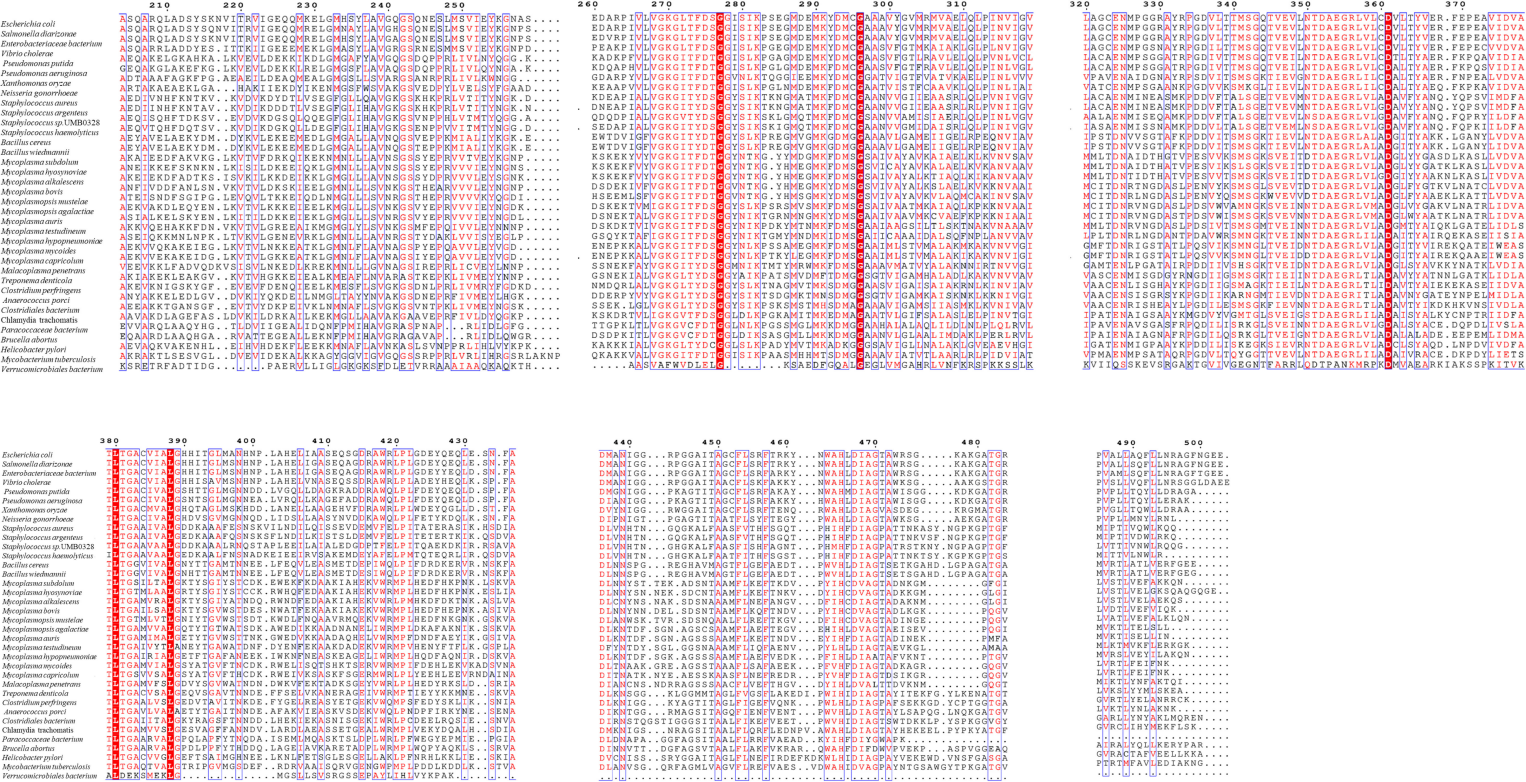
Multiple sequence alignment of M17-LAPs showing conserved catalytic motifs and variable regions. Highly conserved catalytic residues (Asp279, Asp369, Lys389, and Asp399) are highlighted in red. Semi-conserved residues, which have similar chemical properties such as being hydrophobic or acidic, are highlighted in blue. Variable positions are shown in light or blank columns.

**Fig 5:**
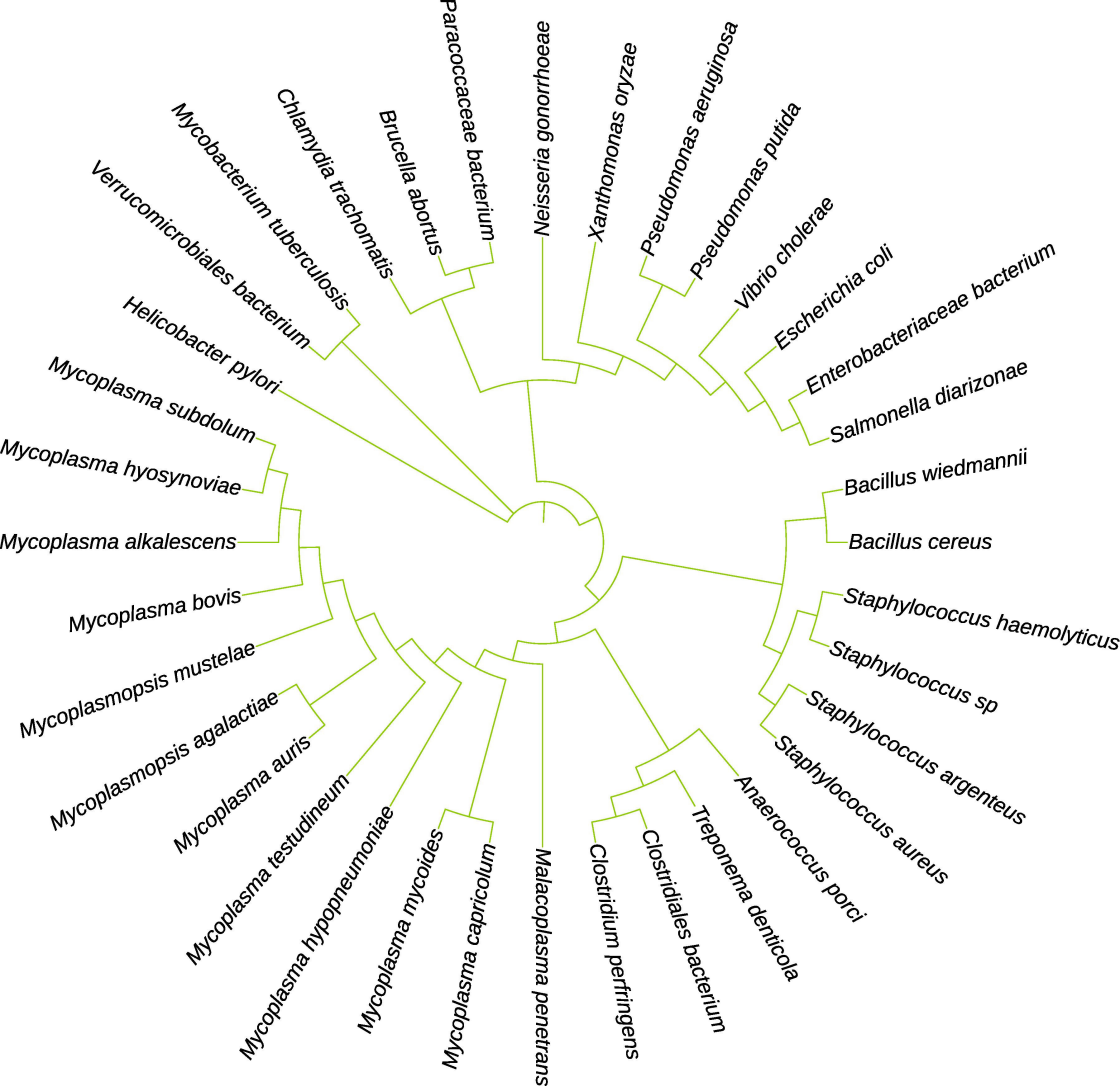
Phylogenetic relationship of the M17-LAPs. An unrooted dendrogram was created by comparing the full-length amino acid sequences of LAP family members using the CLUSTAL W alignment tool. The phylogram was generated in MEGA software after 1,000 bootstrap replicates using the neighbor-joining method. The tree is drawn to scale, with branch lengths measured in the same units as the evolutionary distances used to construct the phylogenetic tree.

A multiple sequence alignment and phylogenetic analysis of M17LAPs from representative bacterial species was aligned using Clustal Omega and visualized in ESPript 3.0 to identify conserved regions and functionally important residues (Fig. 4 and 5). The alignment revealed a high level of sequence conservation within the catalytic core region, characteristic of the M17 family. Several amino acids—Asp279, Asp369, Lys389, and Asp399—were found to be strictly conserved across all sequences. These residues are known to coordinate two metal ions (Zn²^+^/Mn²^+^) that form the binuclear metal center, which is essential for peptide hydrolysis. Conserved glycine-rich motifs near positions 270–280 and 350–370 correspond to the active site signature of the M17 domain, confirming the identity of the proteins. In contrast, variable residues were observed mainly in surface-exposed regions, particularly between amino acid positions 300–340 and 450–480, suggesting species-specific flexibility that may affect substrate access or regulation.

The strong conservation of the metal-binding and catalytic residues (Asp279, Asp369, Lys389, and Asp399) across various bacterial species shows that the catalytic mechanism of M17 LAPs remains evolutionarily stable. These residues create a binuclear metal-binding pocket, a crucial feature of the M17 domain and key to enzymatic activity.

The alignment also shows that, while the active site and core domain are highly conserved, the surrounding loops exhibit structural differences, possibly reflecting adaptation to different substrates or host environments. This combination of conserved catalytic machinery and variable outer regions suggests that the M17 Family keeps its enzymatic function while allowing flexibility for species-specific interactions. From a therapeutic standpoint, preserving these active-site residues across different bacterial species supports the potential to design broad-spectrum inhibitors targeting the M17 LAP Family for fighting bacterial infections.

The biological activities of M17 aminopeptidases linked to target selectivity are mainly caused by their LAP activity. Additionally, certain M17 enzymes, predominantly from bacterial sources, display cysteinyl-glycinase activity, integral to their essential cellular functions. Furthermore, another subset of M17-LAPs performs roles independent of enzymatic activity, relying instead on their quaternary structure, such as in transcriptional regulation (Table 1).

**TABLE 1:** The biological functions and molecular properties of M17-LAPs in bacteria

Bacteria	Protein name	Molecular activity	Main functions	Involvement in virulence	References
*S. aureus*	SaM17-LAP	Cysteinyl-glycinase activity, leucyl-aminopeptidase-like hydrolysis	Regulates peptide turnover and modulates cell wall synthesis and signaling pathways, supporting metabolic adaptation	Contributes to virulence but is not essential for survival	(32)
*T. denticola*	TdM17-LAP	Cysteinyl-glycinase activity	Catalyzes glutathione catabolism via cysteinyl-glycine cleavage, maintaining intracellular redox balance	Not essential, role in metabolic adaptation	(51, 59, 60)
*H. pylori*	HpM17LAP	Arginyl-aminopeptidase and cysteinyl- glycinase activities	Regulates arginine homeostasis, protects against macrophage defenses, supports protein quality control and drug resistance	Essential for survival and virulence	(53, 54, 61–63)
*P. aeruginosa*	PhpA	LAP hydrolytic activity, quaternary enzyme structure	Regulates transcription of *algD*, controlling alginate biosynthesis and biofilm formation	Not essential but supports virulence traits	(46)
*V. cholera*	VcPepA	Leucyl-aminopeptidase activity, quaternary structure	Alters transcription of the cholera toxin gene in response to environmental stress signals	Involved in virulence regulation but not essential	(22)
*M. tuberculosis*	MtLAP	Activity of leucyl-aminopeptidase	Degrades peptides to support nitrogen recycling and persistence in nutrient-limited conditions	Not essential, limited evidence of virulence contribution	(33)
*B. cereus*	BcLAP	Activity of leucyl-aminopeptidase	Removes N-terminal leucine residues, contributes to metabolic flexibility and virulence in host environments	Not essential, supports virulence	(1)
*E. coli*	EcPepA, EcPepB	LAP hydrolytic activity, crystal structure resolved	Multifunctional; regulates transcription and site-specific DNA recombination; participates in DNA repair and regulation	Not essential	(27)
*M. hypopneumoniae*	MhM17-LAP	Leucyl-aminopeptidase activity with heparin-binding capability	Mediates adhesion, enabling host cell invasion and intracellular survival within macrophages	Essential	(27, 55–57)
*M. bovis*	MbM17-LAP	Leucyl-aminopeptidase activity	Acts as an adhesion protein that binds host extracellular matrix components, likely contributes to immune evasion and colonization	Involvement is suspected but not fully defined	S.L. Chen and H. Askar, unpublished data
*P. putida*	PpLAP	Crystal structures solved at different pH, inhibitor binding (Bestatin)	Provides insights into structural flexibility and inhibitor interactions, model for LAP enzyme mechanisms	Not essential	(64)

## THE PRIMARY FUNCTION OF M17 AMINOPEPTIDASES

### M17 aminopeptidases dependent on LAP activity

#### *M. tuberculosis *M17-LAP

Bestatin suppresses the growth of *M. tuberculosis* in both laboratory and macrophage infection conditions, suggesting that MtM17-LAP is implicated in both the metabolic and disease aspects of *M. tuberculosis*. Bacterial survival and pathogenicity *in vivo* are contingent upon this enzyme (33).

#### *M. bovis* M17-LAP

MbM17-LAP is a new adhesion protein that binds to various host extracellular matrix proteins, playing a key role in *M. bovis* attachment to host cells and its spread. These findings reveal an intracellular leucine aminopeptidase M17 that affects disease development in *M. bovis* (Fig. 2).

### M17 aminopeptidases depend on cysteinyl-glycinase

#### *S. aureus* M17-LAP

The pathogenicity of *S. aureus*, although not entirely dependent, is significantly influenced by its M17-LAP (SaM17-LAP). This enzyme is not only crucial but also urgent for the survival of bacteria within human macrophages *in vitro*. Moreover, *S. aureus* with a mutation in the SaM17-LAP gene showed a substantial decrease in virulence in mice models of both localized and systemic infections. Researchers have suggested that SaM17-LAP regulates signaling, metabolism, and cell wall formation by activating or inactivating several proteins. The bacteria likely benefit from this proteolytic activity in the complex host environment (32). Instead of glutathione, *S. aureus* makes bacillithiol (Cys-GlcN-mal), a low-molecular-weight thiol (65). Cysteine-containing compounds are essential for supplying cysteine under nutritional limitations (66) and play a crucial role in defense against low pH, oxidative stress, and osmotic pressure. Furthermore, pathogenicity has been associated with sulfur metabolism (67, 68). The significance of SaM17-LAP for *S. aureus* pathogenicity is shown by its Cys-Gly activity (49).

#### *T. denticola* M17-LAP

The glutathione degradation pathway is expected to be significantly influenced by the TdM17-LAP, an enzyme that catalyzes cysteinyl-glycinase reactions. This organism plays a pivotal role in the process. Immune depletion studies aimed at targeting the soluble fraction of sonicated *T. denticola* cells cultured under standard conditions were used to derive this result. The cells were targeted for their significant cysteinyl-glycinase activity. This pathway yields equimolar amounts of hydrogen sulfide, ammonium, pyruvate, glutamate, and glycine (51, 59). Both glutathione and Cys-Gly play a vital role in preserving the delicate redox balance within cells, safeguarding their integrity from oxidative damage. Moreover, these remarkable thiol-containing compounds can modify cysteine residues in specific proteins, expertly regulating their functions and activities (60).

#### *H. pylori* M17-LAP

HpM17-LAP activation occurs in response to nitric oxide (61) and metronidazole-induced oxidative stress (62). The results indicate that HpM17-LAP has allosteric properties and a high efficiency, which point to its potential involvement in the *H. pylori* life cycle (54). The involvement of HpM17-LAP in *H. pylori* survival is exciting; the response of human macrophages to nitric oxide (61) indicates a protective role (53). In addition to serving as an essential housekeeper, the reaction to metronidazole suggests a role in the processes surrounding drug resistance (52). The cysteinyl-glycinase activity of HpM17-LAP may be the cause of its functions in response to oxidative stress in cells (52, 53). *H. pylori* gets glutamate from stomach mucosal glutathione (63), which protects it from osmotic stress, oxidative damage, and low pH (67). Restoring cysteine requires cleaving the Cys-Gly dipeptide, which is produced during glutathione catabolism (53). On the other hand, HpM17-LAP’s increased activity on peptides with a necessary nitrogen-terminal arginine (69) is crucial to maintain cytoplasmic free arginine levels for the generation of amines, or polyamines, necessary for the development of *H. pylori* (53). Bestatin prevents *H. pylori* from growing *in vitro* (54), due to its suppression of LAP (27).

### The quaternary structure of M17 aminopeptidases is essential to their primary function

#### *P. aeruginosa* M17-LAP

*P. aeruginosa’s* hexameric M17-LAP modulates the virulence-related *algD* gene, which codes for an enzyme in the alginate biosynthesis pathway (PhpA) (46). This novel finding provides previously unknown insights into how alginate contributes to the formation and overproduction of biofilms, which are associated with the extremely mucoid phenotype observed in lungs affected by cystic fibrosis, potentially leading to the development of novel therapeutic approaches (47). Mutating a metal-binding residue of PhpA, unlike inhibition with Bestatin, results in increased *algD* transcription and slow growth *in vivo*, suggesting that transcriptional control is not dependent on aminopeptidase activity (46), and such mutations might result in hexamer disruption (50). The potential implications are likely to spark further investigation and discussion in the field.

#### *V. cholerae* M17-LAP

The synthesis of virulence components, including cholera toxin, in *V. cholerae* is regulated by a complex network that is significantly influenced by environmental temperature and pH. Disruption of the gene encoding M17-LAP in *V. cholerae* (VcPepA) resulted in higher cholera toxin levels, even under non-inducing conditions (pH 8.4 and 37°C), where toxin production typically does not occur. Under inducing conditions (pH 6.5 and 30°C), the lack of VcPepA does not influence toxin levels (22). The discovery of a putative target region for VcPepA binding in the genome of *V. cholerae* by (22) underscores the crucial role of this protein in controlling the transcription of the toxin gene, thereby providing a deeper understanding of how VcPepA responds to changing environmental factors. The enzymatic activity of VcPepA is not implicated in this function.

## POTENTIALS AS A DRUG TARGET

As we deepen our understanding of the various processes mediated by M17-LAPs, we may discern potential applications for these functions or justifications for medically interfering with them. The initial explicit use is the formulation of inhibitors targeting a specific M17-LAP for therapeutic use. The potential of M17-LAPs as therapeutic targets is not only intriguing but also engaging for researchers and scientists. In pathogenic organisms, the ablation of aminopeptidase activity leads to the microorganism’s demise, constituting a straightforward procedure. SaM17-LAP and VcPepA are M17-LAPs that may contribute to virulence in various organisms. However, it is a validated strategy for drug discovery to target the virulence of infectious microorganisms (70). *Fasciola*, a parasitic flatworm that infects both humans and animals, has inspired a new direction in vaccine development. M17-LAPs are under investigation as a potential vaccine candidate, with findings that have been inconsistent. Immunization with *Fasciola* spp. M17-LAP enzymes has elicited a protective immune response in mice (71), sheep (72), and rabbits (13), offering a promising avenue for future vaccine development, even though no such response was detected in buffalo (73). M17-LAPs in microbes can bind DNA and are involved in protein degradation, also known as proteolysis (21). Due to their potential as therapeutic targets and vaccine candidates for numerous parasitic and bacterial diseases, research on members of the M17 family has been heightened recently (13, 15, 16, 32, 74).

Individuals of the M17 family, released via several protozoan and parasite species, are presently the focal point of significant studies as potential therapeutic and preventative medication targets (16, 18, 72). Moreover, the M17 circle of relatives in individuals has been studied in microorganisms, particularly gram-negative microorganisms, which play a role in virulence (22, 25, 31).

In a groundbreaking revelation, researchers have identified a member of the M17 family that plays a crucial role in the virulence of the bacterium. This discovery sheds new light on the mechanisms underlying bacterial pathogenicity, opening the door to potential new avenues for treatment and prevention. This study provides the first evidence that an intracellular aminopeptidase, LAP, affects virulence in a gram-positive bacterium. In both the Newman strain and the USA300 background, disrupting the *pepZ* gene, which encodes LAP, significantly decreased disease severity in both systemic infection models and localized abscess models. The *pepZ* mutant bacteria exhibited a markedly reduced capacity to survive within human macrophages, indicating that this virulence deficiency may be replicated during human infection (32).

Research on *Plasmodium* M17 aminopeptidases has been conducted to explore their potential as novel drug targets for the development of new antimalarial medications (75–78). The investigation of enzyme structure, function, and inhibition as drug targets has revealed that metal ion abundance governs the enzyme’s oligomeric equilibrium, subsequently regulating proteolysis (76, 78, 79).

The potential therapeutic value of aminopeptidase M17 as a drug target in bacteria is particularly evident in bacterial infections. In the context of drug discovery, the following is a strategy for targeting this enzyme.

Understand the crucial function of M17-LAP in peptide hydrolysis and virulence factors in bacterial physiology: M17-LAP helps bacteria recycle peptides and amino acids by catalyzing the removal of amino acids from peptides’ N-terminal. If this enzyme is disrupted, bacteria may be unable to utilize nutrients effectively, which could compromise their ability to grow and survive. Pathogenic bacteria may utilize aminopeptidases in immune evasion, bacterial adhesion, or toxin synthesis mechanisms. Inhibiting M17-LAP may compromise these virulence pathways. Conventional high-throughput screening may be employed to discover small compounds or peptides that selectively inhibit M17-LAPs. This method involves screening extensive libraries of chemicals to ascertain those that interact with the enzyme. Since M17-LAP operates on peptides, developing peptidomimetic compounds that emulate the enzyme’s natural substrate may prove advantageous. These substances may occupy the enzyme’s active site, inhibiting its action. The availability of the crystal structure of M17-LAP facilitates the development of effective and selective inhibitors using a structure-based drug design. *In silico* docking research can forecast the interaction of tiny molecules with the enzyme’s active site.

A significant challenge in using enzyme inhibitors as therapeutic agents is their bioavailability. The development of delivery systems, such as nanoparticles and liposomes, may enhance the stability and absorption of inhibitors, particularly in the context of targeting intracellular bacterial populations. Certain strategies focus on designing drugs specifically targeting bacteria while sparing host tissues, utilizing mechanisms such as antibodies or bacterial surface-binding molecules. Similar to other antibiotics or enzyme-targeting drugs, the evolution of bacterial resistance is possible. Monitoring the development of resistance mechanisms is essential, including mutations in the M17-LAP enzyme that diminish drug binding and alterations in bacterial metabolism that prevent the requirement for this enzyme. Combination therapies involving M17-LAP inhibitors alongside other antibiotics or treatments may diminish the likelihood of resistance development, thereby enhancing the therapy’s long-term efficacy. If inhibiting M17-LAP considerably disrupts bacterial protein homeostasis, it may result in undesirable side effects, such as bacterial cell lysis or an immunological reaction. A significant challenge is ensuring that inhibitors are specific to the bacterial M17-LAP without affecting aminopeptidases.

Targeting aminopeptidase M17 in bacteria for drug development is a promising strategy, especially for treating bacterial infections and addressing antibiotic resistance. By identifying selective inhibitors, developing delivery systems, and evaluating resistance mechanisms, M17-LAP could serve as an effective drug target. However, careful consideration must be given to specificity, resistance, and potential side effects during the development of such therapies.

## CONCLUDING REMARKS

In conclusion, aminopeptidase M17 plays a crucial role in bacterial proteostasis by hydrolyzing amino acid residues from the N-terminus of peptides, thereby regulating cellular activities such as protein maturation and degradation. The enzyme’s highly conserved structure, which includes a catalytic M17 metalloprotease domain, makes it an attractive target for therapeutic development, particularly given its critical role in bacterial growth and survival. The selectivity of M17 enzymes across various bacterial species suggests the possibility of generating selective inhibitors that could function as new antimicrobial agents, particularly in light of the escalating antibiotic resistance. Furthermore, understanding the structure–function relationship of these enzymes opens up new possibilities for optimizing therapeutic strategies and increasing the efficacy of existing treatments. Future studies on the structural biology of aminopeptidase M17 and its interactions with other cellular components will be crucial for developing more targeted and effective antibacterial therapies. Further study is required to understand how M17-LAP affects bacterial pathogenicity fully and to investigate whether it may be used as a potent vaccine for certain bacterial diseases. Although M17-LAP is being investigated as a potential vaccine for parasitic disorders, its application as a vaccine against bacteria is a less well-studied field of research.

## Data Availability

The authors confirm that the data supporting the findings of this study are available within the article.
